# Short-term efficacy of right-to-left shunt closure in patients with vestibular migraine

**DOI:** 10.3389/fneur.2024.1500918

**Published:** 2024-12-12

**Authors:** Yilin Lang, Sai Zhang, Peifan Xie, Yang Wang, Chuangwei Wang, Wenting Wang, Xien Zhu, Ping Gu

**Affiliations:** Department of Neurology, The First Hospital of Hebei Medical University, Shijiazhuang, China

**Keywords:** vestibular migraine, right-to-left shunt closure, efficacy, patent foramen ovale, pulmonary arteriovenous malformation

## Abstract

**Objective:**

This study aims to evaluate the short-term efficacy of right-to-left shunt closure in vestibular migraine patients, and compare the efficacy between patent foramen ovale (PFO) closure and pulmonary arteriovenous malformation (PAVM) embolization. Additionally, the study identifies factors related to surgical outcomes.

**Methods:**

Forty-one patients with vestibular migraine and medium to large right-to-left shunts underwent surgery: PFO closure, PAVM embolization, or both. Baseline data and postoperative outcomes at one month, including migraine and dizziness frequency, duration, VAS, HIT-6, migraine scores, and DHI scores, were analyzed. The correlation between efficacy and baseline data was analyzed.

**Results:**

At one month postoperatively, the frequency and duration of dizziness and migraine attacks significantly decreased, and the VAS, HIT-6, migraine scores, and DHI scores all significantly dropped (*p* < 0.001). There was no significant difference in the improvement rates of VAS, HIT-6, migraine scores, and DHI scores between the PFO group and the PAVM group (*p* > 0.05). Red blood cell parameters positively correlated with the improvement rates of VAS, HIT-6, migraine scores, and DHI. This approach was more effective in male patients than in female patients (*p* < 0.05).

**Conclusion:**

Right-to-left shunt closure has a significant short-term effect on patients with vestibular migraine, regardless of the shunt location. Red blood cell parameters may serve as predictive indicators for the surgical efficacy in these patients.

## 1 Introduction

Vestibular migraine (VM) is the second most common cause of episodic vertigo, with a prevalence of approximately 1–2.7% in the general population (1). VM is primarily characterized by the temporal association of migraine symptoms and vestibular symptoms, which may be accompanied by photophobia, phonophobia, nausea, and vomiting. These recurrent symptoms severely impact the quality of life of patients. As a relatively new diagnostic entity, the pathophysiological mechanisms and treatment approaches for VM are largely based on migraine research. Previous studies have suggested a possible correlation between migraine, VM, and right-to-left shunt (RLS) (2).

RLS is an abnormal hemodynamic change where blood that has not been fully oxygenated in the lungs passes directly from the right heart system to the left heart system due to structural cardiovascular abnormalities. RLS is mainly found in patent foramen ovale (PFO) and pulmonary arteriovenous malformation (PAVM). The relationship between PFO and migraine has been a research hotspot in recent years. Although there is a lack of high-quality evidence supporting a causal relationship, some hypotheses partially explain the role of PFO in migraine. For example, PFO may cause microemboli in the systemic circulation to enter the arterial system directly, subsequently reaching the brain and causing paradoxical embolism. In the presence of RLS, deoxygenated venous blood enters the arterial blood, potentially leading to brain tissue hypoxia. These mechanisms may trigger cortical spreading depression, which in turn leads to migraine attacks (3–6). While the pathophysiological mechanisms of PFO and PAVM are not entirely identical, the aforementioned mechanisms can also occur in PAVM (7).

Numerous studies have shown that PFO closure can alleviate migraine symptoms, and PAVM embolization also benefits migraine patients (8). Although there is controversy regarding the efficacy and necessity of surgery, many studies have reported significant relief of migraine symptoms in some patients (3, 8, 9). Therefore, rather than discussing the necessity of surgery, further optimizing the surgical indications may be more important. The treatment of VM lacks randomized controlled trials, and the quality of evidence from related studies is relatively low. However, existing research supports that many migraine treatments are effective for VM (10). Based on this, it is hypothesized that RLS closure is also likely effective for some VM patients. It is necessary to further explore the efficacy of RLS closure in VM patients and identify those who are more likely to benefit from the surgery.

We conducted a study with a small sample size to observe the short-term efficacy of RLS closure in VM patients. The results of this study will provide preliminary findings and new perspectives for future research on VM combined with RLS.

## 2 Methods

### 2.1 Study design

This study utilized a prospective cohort study design and was approved by the Ethics Committee of the First Hospital of Hebei Medical University. The study included 41 VM patients with medium to large RLS who were scheduled for interventional surgery and visited the First Hospital of Hebei Medical University from October 2023 to March 2024.

Inclusion criteria: Patients met the diagnostic criteria for VM as defined by the third edition of the International Classification of Headache Disorders (ICHD-3); aged 16–60 years; signed informed consent.

Exclusion criteria: Other diseases causing migraine and dizziness; patients with mental disorders or cognitive impairment.

### 2.2 Study methods

#### 2.2.1 Observational indicators

Preoperatively, baseline data were recorded for the patients, including general information like gender, age, BMI, medical history, or other comorbidities. The clinical characteristics of VM before surgery were also collected, such as disease course, relationship between migraine and dizziness, frequency and duration of migraine and dizziness attacks, Visual Analog Scale (VAS) scores, Headache Impact Test (HIT-6) scores, migraine scores, Dizziness Handicap Inventory (DHI) scores, presence of triggers, and accompanying symptoms. Hematological indicators were also recorded, including red blood cell count (RBC), hemoglobin (HGB), hematocrit (HCT), platelet count (PLT), mean platelet volume (MPV), plateletcrit (PCT), fibrinogen (FIB), D-dimer (DD), and fibrinogen degradation products (FDP). The RLS shunt volume was assessed and defined as: no shunt, small (saline contrast microbubbles <10), medium (saline contrast microbubbles 10–30), large (saline contrast microbubbles >30), and very large (left atrial cloudiness).

#### 2.2.2 Surgical methods

Based on angiographic results and intraoperative findings, patients underwent PFO closure and/or PAVM embolization. All patients were prescribed oral aspirin and clopidogrel for 6 months postoperatively.

### 2.3 Efficacy follow-up

At one month after surgery, all patients were evaluated for the occurrence of migraine and dizziness. For patients who still experienced attacks, the frequency and duration of attacks, as well as VAS, HIT-6, migraine scores, and DHI scores, were recorded. The improvement rates for VAS, HIT-6, migraine scores, and DHI scores were calculated, with the HIT-6 improvement rate calculated by subtracting the baseline score of 36 from the HIT-6 score.

### 2.4 Statistical methods

SPSS 25.0 statistical software was used for data analysis. Relevant score data were presented as medians (interquartile ranges) and analyzed using rank-sum tests. Categorical data were presented as percentages and analyzed using the χ2 test or Fisher’s exact test. Correlations between variables were assessed using Spearman’s correlation analysis. A *p*-value of <0.05 was considered statistically significant.

## 3 Results

### 3.1 Clinical baseline data

This study included a total of 41 patients with VM combined with medium to large RLS, aged between 16 and 60 years, with a male-to-female ratio of 1:3.1. Among all patients, 3 had a history of stroke, 4 had a history of hypertension, 1 had a history of hyperlipidemia, and 1 had a history of anemia. The remaining patients had no other relevant medical history.

According to the surgical procedures received by the patients, they were divided into 3 groups: PFO group (25 cases), PAVM group (14 cases), and PFO + PAVM group (2 cases). Comparative analysis of baseline data between the PFO and PAVM groups showed that, except for higher HIT-6 scores in the PFO group compared to the PAVM group, there were no significant differences in other baseline data between the two groups (*p* > 0.05). Specific data are detailed in Table 1.

**Table 1:** Comparison of Baseline Data between PFO Group and PAVM Group.

	PFO group (*n* = 25)	PAVM group (*n* = 14)	*P*
Age (years)	37 (17.5–44.5)	32.5 (16.75–37.5)	0.186
Gender (*n*, %)			
Male	6 (24.0)	3 (21.4)	1.000
Female	19 (76.0)	11 (78.6)	
BMI (kg/m^2^)	20.76 (19.41,24.48)	22.58 (19.98,24.57)	0.429
Duration of Migraine (years)	4 (2.5,10)	3 (0.92,7.75)	0.217
Duration of Dizziness (years)	2 (0.5,6)	2 (0.63,4.25)	0.780
Migraine frequency (*n*, %)			
≤5 /month	13 (52)	7 (50)	1.000
>5 /month	12 (48)	7 (50)	
Migraine frequency (*n*, %)			
≤5/ month	9 (36)	7 (50)	0.503
>5/ month	16 (64)	7 (50)	
Migraine duration (*n*, %)			
Within few hours	5 (20)	2 (14.3)	0.089
1 day	15 (60)	8 (57.1)	
Few days	5 (20)	4 (28.6)	
Dizziness duration (*n*, %)			
Within 1 min	4 (16)	1 (7.1)	0.646
Within few hours	9 (36)	4 (28.6)	
1 day	7 (28)	7 (50)	
Few days	5 (20)	2 (14.3)	
VAS	7 (6,8.5)	5.5 (4,7)	0.055
HIT-6	64 (57.5,69)	59 (52.25,62.25)	0.046
Migraine score	14 (11,15.5)	13 (10.75,14.5)	0.289
DHI	30 (16,53)	27 (11.5,39.5)	0.395
Presence of triggers (*n*, %)			
Yes	17 (68.0)	12 (85.7)	0.279
No	8 (32.0)	2 (14.3)	
Relationship between migraine and dizziness (*n*, %)			
Migraine precedes dizziness	20 (80.0)	7 (50.0)	0.062
Dizziness precedes migraine	4 (16.0)	3 (21.4)	
Simultaneous occurrence	1 (4.0)	4 (28.6)	
Presence of aura (*n*, %)			
Yes	7 (28.0)	3 (21.4)	0.721
No	18 (72.0)	11 (78.6)	
Associated symptoms			
Photophobia and/or phonophobia			
Yes	16 (64.0)	7 (50.0)	0.503
No	9 (36.0)	7 (50.0)	
Nausea and vomiting			
Yes	21 (84.0)	12 (85.7)	1.000
No	6 (16.0)	2 (14.3)	
Other symptoms			
Yes	18 (72.0)	10 (71.4)	1.000
No	7 (28.0)	4 (28.6)	
Diversion volume			
Moderate	3 (12.0)	4 (28.6)	0.203
Large	15 (60.0)	9 (64.3)	
Very large	7 (28.0)	1 (7.1)	

### 3.2 Comparison of changes in dizziness and migraine before and after surgery

**Figure 1:**
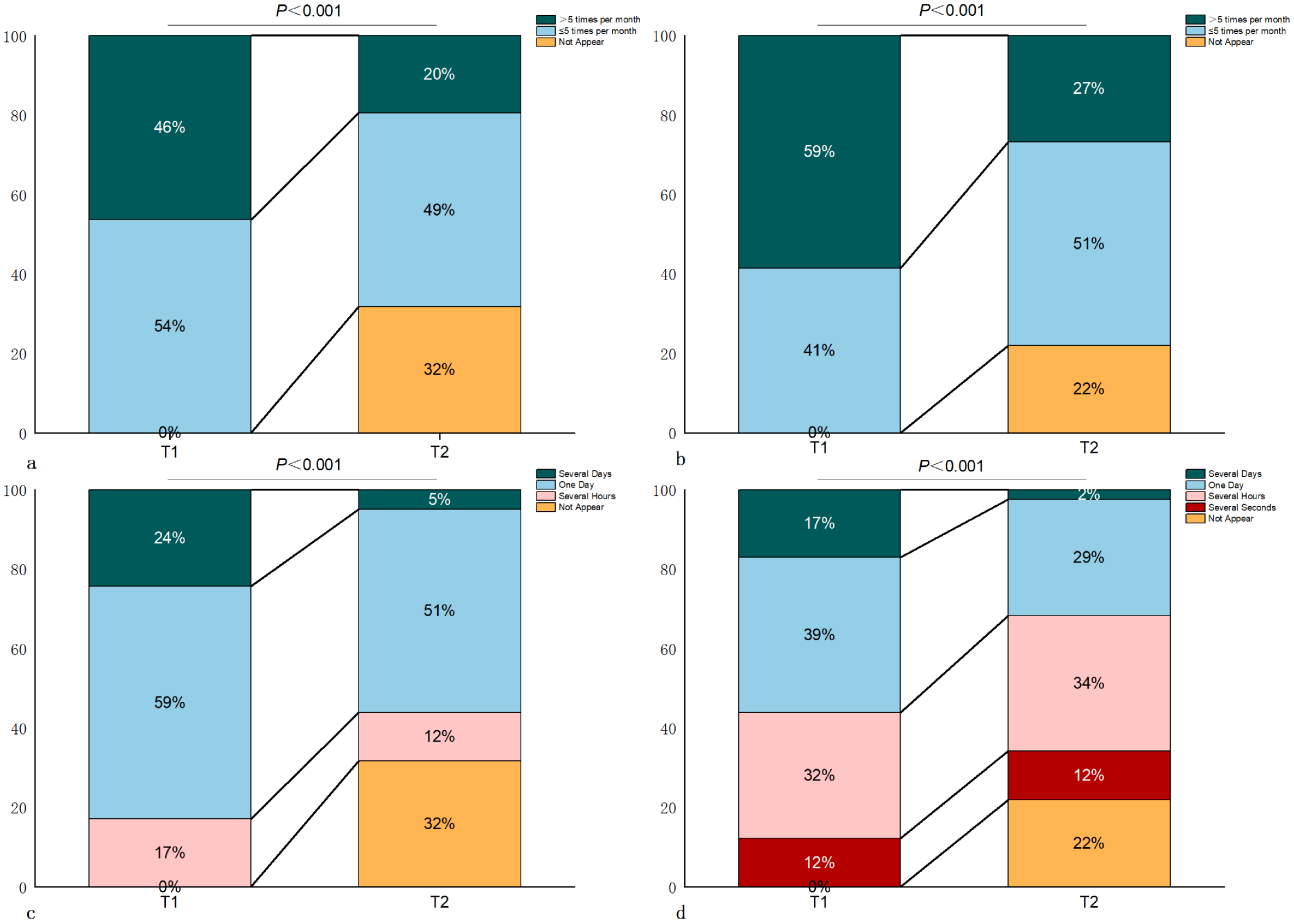
Changes in frequency and duration of migraine and dizziness in VM patients. (Note: a. Frequency of migraine in VM patients; b. Frequency of dizziness in VM patients; c. Duration of migraine in VM patients; d. Duration of dizziness in VM patients. T1 - preoperative; T2 - 1 month postoperative).

At one month postoperatively, the frequency of dizziness and migraine attacks in patients significantly decreased, with significantly shortened duration per attack (both *p* < 0.001). Specifically, 13 patients (32%) did not experience migraine attacks at one month after surgery, and 9 patients (22%) did not experience vertigo attacks postoperatively (Figure 1).

Additionally, in patients with VM combined with RLS, at one month after occlusion surgery, VAS, HIT-6, migraine scores, and DHI scores all significantly decreased (*p* < 0.001). Specific data are shown in Table 2.

**Table 2:** Comparison of severity of migraine and dizziness in VM patients before and 1 month after surgery [M (P25, P45)].

	T1	T2	P
VAS	7 (5,8)	2 (0,3.75)	<0.001
HIT-6	61 (55,67)	50 (36,56.5)	<0.001
migraine scores	13 (11,15)	7 (0,11)	<0.001
DHI	28 (14,45)	14 (3,27)	<0.001

T1 - preoperative; T2–1 month postoperative.

### 3.3 Comparison of efficacy between PFO closure and PAVM embolization

At one month postoperatively, there were no significant differences between the PFO group and the PAVM group in terms of postoperative frequency and duration of migraine, as well as frequency and duration of dizziness (*p* > 0.05).

**Figure 2:**
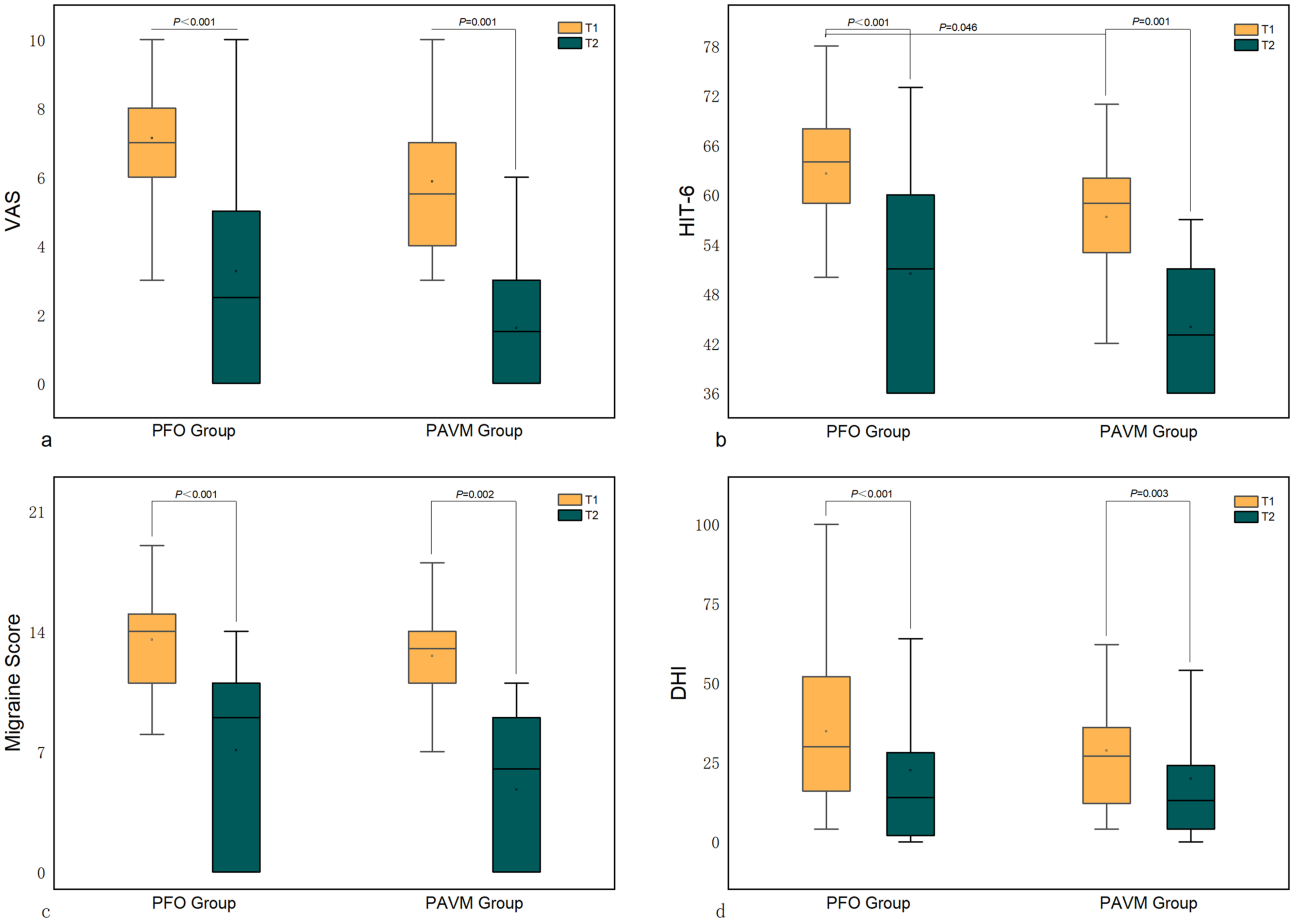
Comparison of scores between the PFO group and the PAVM group postoperatively. (Note: T1 - preoperative; T2 - 1 month postoperative).

Both the PFO group and the PAVM group showed significant reductions in VAS, HIT-6, migraine scores, and DHI scores at one month postoperatively compared to preoperative values (*p* < 0.001). However, there were no significant differences between the two groups in terms of VAS, HIT-6, migraine scores, and DHI scores one month after surgery. Despite the higher preoperative HIT-6 score in the PFO group compared to the PAVM group, there were no significant differences in the improvement rates of VAS, HIT-6, migraine scores, and DHI scores between the two groups (*p* > 0.05) (Figure 2; Table 3).

**Table 3:** Comparison of efficacy between the PFO group and the PAVM group at 1 month postoperatively [M (P25, P45)].

	PFO group (*n* = 25)	PAVM group (*n* = 14)	*P*
Improvement rate in VAS	0.57 (0.28,1)	0.7 (0.57,1)	0.189
Improvement rate in HIT-6	0.39 (0.15,1)	0.48 (0.29,1)	0.482
Improvement rate in migraine scores	0.39 (0.24,1)	0.48 (0.21,1)	0.561
Improvement rate in DHI	0.25 (0.12,0.77)	0.21 (0.14,0.63)	0.988

### 3.4 Correlation analysis of postoperative efficacy in VM patients with RLS

**Figure 3:**
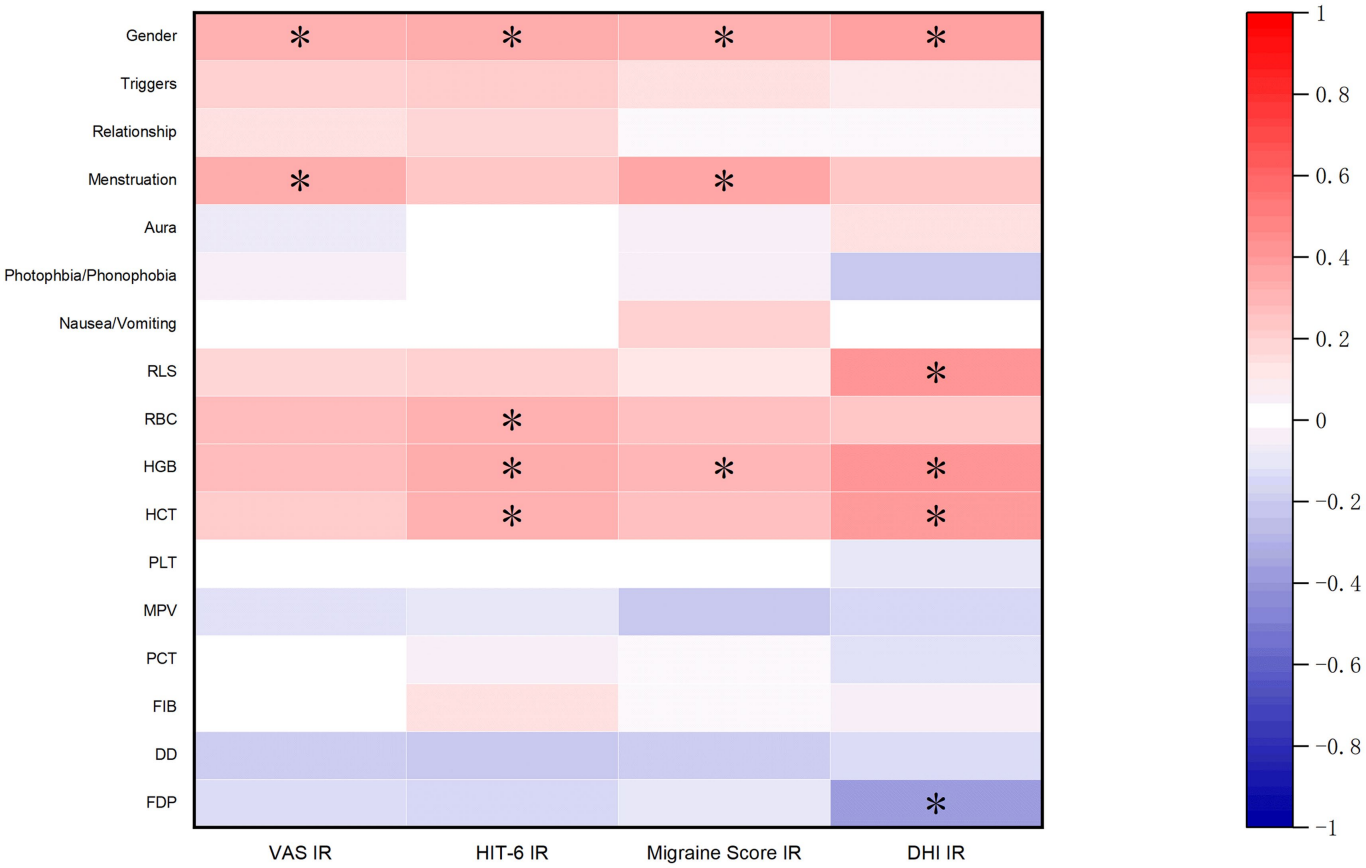
Factors related to surgical efficacy. (Note: *p < 0.05; IR: Improvement Rate).

This study analyzed the correlation of patients’ general information, clinical characteristics, and hematological parameters with the improvement rates of VAS, HIT-6, migraine scores, and DHI scores. The results showed that the improvement of migraine and dizziness symptoms was related to gender. The improvement rates of VAS and migraine scores were related to whether the symptoms worsened during the menstrual period. RBC, HGB, and HCT levels were positively correlated with the improvement rates of VAS, HIT-6, migraine scores, and DHI scores. Specifically, the improvement rate of HIT-6 was statistically significantly correlated with RBC, HGB, and HCT; the improvement rate of migraine scores was significantly correlated with HGB; and the improvement rate of DHI was significantly correlated with HGB and HCT (*p* < 0.05). Additionally, the improvement rate of DHI was positively correlated with RLS and negatively correlated with FDP (Figure 3).

Further comparisons were made between different genders, female patients with or without symptom exacerbation during menstruation, and RLS shunt volume. It was found that the improvement in migraine and dizziness symptoms after surgery was more significant in male patients than in female patients (*p* < 0.05). There was no significant difference in the improvement rates between female patients with and without exacerbation of symptoms during menstruation (*p* > 0.05). VM patients with a very high volume of RLS had a higher improvement rate in migraine and dizziness scores postoperatively compared to those with moderate and high volumes of shunt. However, only the improvement rate in DHI between moderate and very high shunt volumes was statistically significant. Specific data are shown in Table 4.

**Table 4:** Factors related to surgical efficacy [M (P25, P45)].

	Improvement rate in VAS	Improvement rate in HIT-6	Improvement rate in migraine scores	Improvement rate in DHI
Sex				
Male	1 (0.52,1)	1 (0.38,1)	1 (0.35,1)	1 (0.16,1)
Female	0.57 (0.28,0.8)*	0.33 (0.14,0.5)*	0.37 (0.2,0.54)*	0.17 (0.04,0.35)*
Exacerbation during menstruation (female)				
Yes	0.63 (0.2,1)	0.33 (0.07,1)	0.42 (0.26,1)	0.14 (0,0.3)
No	0.54 (0.28,0.63)	0.31 (0.17,0.49)	0.34 (0.15,0.44)	0.19 (0.13,0.46)
Shunt volume				
Moderate	0.54 (0.18,0.95)	0.33 (0.08,0.87)	0.39 (0.04,0.88)	0.14 (−0.02,0.28)
High	0.6 (0.31,1)	0.39 (0.15,1)	0.36 (0.25,1)	0.17 (0.13,0.35)
Very high	0.71 (0.57,1)	0.63 (0.31,1)	0.5 (0.21,1)	1 (0.32,1)#

*Compared to males, *P* < 0.05; #Compared to moderate shunt volume; *P* < 0.05.

There were no significant differences in preoperative VAS, HIT-6, migraine scores, and DHI scores between male and female patients (*p* > 0.05). Further analysis of gender-related factors revealed that preoperative RBC, HGB, and HCT levels were significantly lower in female patients than in male patients, while DD levels were higher in female patients (*p* < 0.05).

## 4 Discussion

Our study results indicate that eliminating RLS can significantly alleviate or eliminate symptoms in VM patients, demonstrating significant short-term efficacy in some patients, which is consistent with previous studies on PFO closure treatment for VM (11–13). Although some studies have found a higher incidence of PAVM in VM patients (2) and that PAVM embolization can reduce migraine and improve oxygenation (8, 14, 15), there is currently no specific in-depth research on the relationship between PAVM and VM.

This study has revealed for the first time the efficacy of PAVM embolization in VM patients with concomitant PAVM, and compared the efficacy of PFO closure and PAVM embolization for VM. The results show no significant difference between the two groups. This finding suggests that the surgical efficacy in VM patients may not be related to the site of RLS occurrence, and PAVM embolization may alleviate VM through mechanisms similar to PFO closure, although further research is needed to explore this mechanism.

This study found significant improvement in VM symptoms after RLS closure, but only 22% of patients experienced complete relief, with some patients showing no significant improvement or even worsening of symptoms. This may be related to postoperative thrombus formation and detachment on the closure device or coil surface (11), although all 41 patients received aspirin and clopidogrel antiplatelet therapy postoperatively. Previous studies have reported occurrences of migraine after ASD closure in patients with no history of migraine, and migraine self-improving or resolving within 6 to 12 months, which suggests this mechanism (16, 17). However, thrombus attachment and detachment on the closure device surface may not fully explain all reasons. The pathological mechanisms in VM patients with concurrent RLS are complex, and the long-term recurrence of RLS may induce compensatory or pathological changes in the body, which may not immediately recover postoperatively. Furthermore, although the mechanisms of VM are not fully understood, it is known to involve multiple factors (18). No single hypothesis can completely explain the pathophysiology of all VM patients, even when discussing only the small subset of VM patients with concurrent RLS. The presence of RLS does not necessarily imply a causal relationship with VM; RLS is also highly prevalent in the general population, and most individuals with RLS do not develop VM. Conversely, there are VM patients without RLS, indicating that other mechanisms besides RLS may also contribute to migraine in these patients. Therefore, further exploration of the pathophysiological mechanisms of VM is crucial for improving surgical outcomes and understanding individual differences among VM patients.

Furthermore, this study only explores the short-term efficacy following RLS closure, and there remains insufficient evidence supporting the long-term efficacy of VM closure therapy. Studies have reported that some migraine patients undergoing PFO closure continue to experience persistent migraine symptoms in the long term, and three clinical trials—MIST, PRIMA, and PREMIUM—failed to meet their primary endpoints regarding PFO closure treatment for migraine (19). The feasibility of closure therapy in VM patients is thus inevitably questioned. Further long-term follow-up studies are needed to confirm the long-term efficacy and safety of closure therapy in VM patients. However, both our study and previous research have observed significant post-operative benefits in some patients. Therefore, rather than unequivocally affirming or negating the efficacy of surgery, optimizing surgical indications and selecting patients who are more likely to benefit may be a more crucial endeavor.

The relationship between migraine and red blood cell parameters has garnered significant attention. Numerous studies have explored the connection between migraine and anemia. For instance, research has found that iron deficiency anemia is more common among migraine patients (20), with lower hemoglobin levels observed, particularly in patients with aura migraine and menstrual-related migraine (21). In children with sickle cell disease, low hemoglobin is also associated with the occurrence of migraine (22). However, in non-anemic patients, the relationship between migraine and red blood cell parameters is the opposite; some studies have emphasized the correlation between increased HGB and HCT levels and migraine (23, 24). In a population-based study on female patients, those with lower HGB levels were less likely to experience migraine (25).

This study is the first to investigate the relationship between red blood cell parameters and the efficacy of RLS closure in VM patients. It was found that higher preoperative levels of RBC, HGB, and HCT were associated with a higher rate of improvement in migraine and dizziness severity, although the causal relationship and underlying mechanisms remain unclear.

Hypoxia is closely related to migraine (26, 27). When RLS is present, deoxygenated venous blood enters the arterial system, causing the body to experience a hypoxic state. During hypoxia, ATP production decreases, the function of Na + -K + -ATPase on the cell membrane is reduced, resulting in increased intracellular Na + and decreased K+, which lowers the membrane potential, making it easier to trigger migraine. A similar mechanism might exist in VM. Studies on migraine have found that PFO closure alleviates the hypoxic state (28). Under hypoxia, RBC, HGB, and HCT levels increase compensatorily. That is, high RBC, HGB, and HCT levels may be indicative of higher hypoxia levels; thus, RLS plays a more significant role in such patients, potentially leading to better surgical outcomes. This hypoxia mechanism can also explain the opposite relationship between HGB and migraine in anemic versus non-anemic patients. Based on these mechanisms, we speculate that RLS closure surgery might reduce VM symptoms by improving hypoxia and restoring the lowered threshold. However, there are numerous triggering factors for migraine and VM, including changes in ovarian hormone secretion, weather changes, and diet (29, 30). When these triggers reach a specific threshold, migraine or VM attacks can occur. This implies that even after improving hypoxia, other triggers might still reach the threshold, potentially explaining the varying degrees of postoperative relief.

The relative elevation of RBC, HGB, and HCT not only reflects hypoxic conditions but may also indicate increased blood viscosity (24), which is associated with a higher risk of thrombosis. The incidence of *in situ* thrombosis in PFO is significantly higher in migraine patients than in asymptomatic PFO patients (31), suggesting a close relationship between thrombosis and migraine. The presence of RLS allows microemboli to directly enter the arterial system and subsequently reach the brain, causing paradoxical embolism, which can be prevented by RLS closure. In our results, DD and FIB showed differences at various stages of analysis, supporting the possibility of a prothrombotic state in VM patients with RLS. Patients with low preoperative levels of RBC, HGB, and HCT have a lower risk of thrombosis, and paradoxical embolism may not be the primary mechanism of VM in these patients, thus explaining the differing efficacy observed in our study.

Differences in red blood cell parameters might also result from RLS. Studies have shown that migraine patients experience increased oxidative stress and reduced antioxidant capacity (32–34). The LEARNER study found that HGB and HCT levels in patients with aura migraine and PFO were lower than normal, returning to physiological levels after PFO closure, along with the restoration of antioxidant capacity, supporting the pathogenic role of oxidative stress (35). However, oxidative stress damage is not exclusive to migraine patients with RLS, as there are other pathways leading to oxidative stress damage in migraine patients (36), which might also be the case in VM. Nevertheless, oxidative stress damage does not adequately explain the observed positive correlation between RBC, HGB, HCT levels, and the improvement of VM symptoms, nor the previously reported association between high hemoglobin levels and migraine (23, 37). Expanding the sample size may be necessary to confirm the relationship of red blood cell parameters with migraine and VM.

In our correlation analysis results, significant differences in efficacy between genders were observed in addition to red blood cell parameters. Comparing the baseline data of male and female patients, women had significantly lower levels of RBC, HGB, and HCT than men. This result aligns with the influence of sex hormones on red blood cell parameters but may indicate that our previous hypotheses about the pathophysiological mechanisms between red blood cell parameters, RLS, and VM are not comprehensive enough. Previous studies in migraine patients have also reported that female subjects had lower RBC, HGB, and HCT levels compared to males (24). Women are significantly more affected by migraine and VM than men, a phenomenon that has not been well explained (1, 38, 39), possibly related to differences in sex hormones (40). However, the relationship between sex hormones, red blood cell parameters, and VM is not yet clear. RBC, HGB, and HCT may merely reflect differences in sex hormones, or sex hormones might influence VM pathophysiology through these red blood cell parameters, which requires further research to confirm.

In conclusion, despite the unclear mechanisms, RBC, HGB, and HCT could be promising predictors of surgical efficacy in VM patients with RLS. Future studies should further explore the relationship between these indicators and the clinical characteristics and treatment response in VM patients, aiming to provide more valuable predictive information for the efficacy of RLS surgery in VM patients.

There is still controversy over whether migraine and VM are different stages of the same disease or distinct conditions. Our study results, showing the consistency of improvement in both migraine and vestibular symptoms following RLS closure, suggest the possibility of a common mechanism underlying these symptoms. However, unlike VAS, HIT-6, and migraine scores for the evaluation of migraine symptoms, there was a significant correlation between the improvement rate of DHI scores (used to evaluate vestibular symptoms) and the degree of RLS shunting. The reason for this finding remains unclear and may result from mechanisms independent of migraine that cause vestibular symptoms, or it could be due to factors such as a small sample size or improper grouping of observational indicators, leading to results without clinical significance. This needs further verification in future studies.

Platelet activation and aggregation play a significant role in the occurrence of migraine (41, 42). Some studies have reported the predictive value of platelet parameters for the improvement of migraine following PFO closure (43). However, in this study, no correlation was found between platelet parameters and the efficacy of RLS closure, which may be due to a small sample size, inappropriate selection of platelet parameters, or even differences in the mechanisms of VM and migraine. Additionally, antiplatelet therapy has been reported in previous studies to alleviate migraine symptoms, and the effects of the medication might have exaggerated our efficacy results.

The efficacy of RLS closure in VM patients remains controversial, and the risks associated with the surgery are also a cause for concern. Our study has preliminarily confirmed the efficacy and safety of the surgery. This exploratory work will lay the groundwork for future larger-scale studies and provide new evidence to support the selection of treatment for these patients.

However, in terms of research design, our current study still has limitations. Firstly, our sample size is small, which may limit the generalizability and reliability of the conclusion. Secondly, although our study is focused solely on investigating short-term outcomes, for a condition that is episodic in nature, a one-month follow-up period might still be considered relatively short. The current results may not fully capture the full efficacy of the surgery. Thirdly, this study only used a before-and-after control without establishing a non-surgical observation group or a sham surgery group, and the results may have been influenced by the placebo effect. Additionally, our study only collected baseline data before surgery, lacking the use of a structured symptom diary for baseline symptom tracking, which increases the risk of recall bias. These inherent limitations render the findings of this study exploratory in nature, with limited generalizability. We look forward to future studies that can validate and expand upon our preliminary findings.

Our team plans to extend the follow-up period to include the discontinuation of antiplatelet medications and add a non-surgical antiplatelet medication control group, in order to verify the reliability of the current results. Expanding the study scale is also necessary. Moreover, analyzing the differences between VM and migraines is also necessary to gain a deeper understanding of their pathophysiological mechanisms.

## 5 Conclusion

This study confirmed the significant short-term efficacy of RLS closure surgery in VM patients, regardless of the RLS origin. The results support the role of RLS in the pathophysiology of VM, while RBC, HGB, and HCT levels may serve as predictive indicators of surgical efficacy in VM patients with RLS. In summary, this study provides new insights for the treatment and research of VM, but larger-scale studies are needed to confirm the reliability of these results and to clarify the optimal indications and long-term efficacy of RLS closure surgery.

## Data Availability

The original contributions presented in the study are included in the article/supplementary material, further inquiries can be directed to the corresponding author.
